# Vanadium dioxide-assisted broadband tunable terahertz metamaterial absorber

**DOI:** 10.1038/s41598-019-42293-9

**Published:** 2019-04-08

**Authors:** Huan Liu, Zhi-Hang Wang, Lin Li, Ya-Xian Fan, Zhi-Yong Tao

**Affiliations:** 1 Key Lab of In-fiber Integrated Optics, Ministry Education of China, Harbin Engineering University, Harbin, 150001 People’s Republic of China; 2 Academy of Marine Information Technology, Guilin University of Electronic Technology, Beihai, 536000 People’s Republic of China

## Abstract

Tunable terahertz (THz) functional devices have exhibited superior performances due to the use of active materials, such as liquid crystals, graphene, and semiconductors. However, the tunable range of constitutive parameters of materials is still limited, which leads to the low modulation depth of THz devices. Here, we demonstrate a broadband tunable THz absorber based on hybrid vanadium dioxide (VO_2_) metamaterials. Unlike other phase change materials, VO_2_ exhibits an insulator-to-metal transition characteristic and the conductivity can be increased by 4–5 orders of magnitude under external stimulus including electric fields, optical, and thermal pumps. Based on the unique transition character of VO_2_, the maximum tunable range of the proposed absorber can be realized from 5% to 100% by an external thermal excitation. Meanwhile, an absorption greater than 80% in a continuous range with a bandwidth about 2.0  THz can be obtained when VO_2_ is in its metal phase at high temperature. Furthermore, the absorber is insensitive to the incident angle up to 50° and such a broadband THz absorber can be used in applications including imaging, modulating, cloaking, and so on.

## Introduction

Terahertz (THz) wave lies in the frequency gap between microwave and the infrared, and the corresponding wavelength ranges from 3 mm to 30 μm. Because of its great potential in communication, biomedicine, security, and so on^1–3^, THz technology has attracted more and more attention in recent decades. The key problem affecting the practical application of THz technology is the lack of functional devices with excellent performance. This is mainly due to the absence of materials in nature that can directly interact with THz waves. Fortunately, an artificial composite material consisting of the periodically arranged structural units called the metamaterial (MM) has emerged to solve this problem. Unlike natural materials, MMs can achieve some abnormal electromagnetic (EM) properties by adjusting the resonant unit structure, such as negative refractive index^4^, perfect lensing^5^, invisibility^6^, perfect absorption^7^, and EM induced transparency (EIT)^8^. Because of their unique EM properties, MMs have also been used in THz functional devices, such as modulators^9^, filters^10^, and optical switches^11^.

MM absorbers (MMAs) have great application value in stealth, detection, communication and other fields, and have gradually become one of the research hotspots in THz fields. In 2008, Landy *et al*. first proposed an MMA with perfect absorption characteristics^7^. Subsequently, various MMAs working in different frequency bands were realized in turn, including microwaves^12,13^, THz waves^14,15^, visible and infrared bands^16,17^. In THz band, Tao *et al*. designed and manufactured the first narrowband absorber^18^. Thereafter, dual-band^19^, triple-band^20^, and multi-band THz absorbers^21^ were reported in succession. However, due to the strong dispersion of resonant structures of the early MMA, the broadband THz absorbers were greatly limited. The multi-resonator cascade and dispersion control^22–24^ have also been proposed to extend the absorption bandwidth of MMAs. In these absorbers, the performances have been fixed according to the designed structures, which still limits their practical applications. Thus, it is urgently needed that the active regulation and control of MMAs can be realized by means of heat, electricity, and light.

Recently, researchers have employed some functional materials to achieve tunable THz MMAs. For example, Padilla first demonstrated the electrically tunable THz absorbers based on hybrid liquid crystal MM structures in 2013^25^. In 2017, by combining a planar metal disk resonator with liquid crystal, Wang at al. realized a triple-band tunable THz MMA with sub-wavelength thickness^26^. In the same year, by utilizing the hybrid metasurface structure composed of graphene and gold, Zhao *et al*. achieved excellent THz absorption spectra (0.53–1.05 THz) with wide incident angles for both TE and TM waves^27^. Meanwhile, graphene-assisted highly tunable liquid crystal THz MMA was also demonstrated^28^. However, the tunable range of the constitutive parameters of the graphene and liquid crystal materials is limited, which leads to the low modulation depth of absorbers.

The THz response of vanadium dioxide (VO_2_) is essentially different from these of graphene and liquid crystal. As a photoelectric functional material with high quality, VO_2_ is a kind of metal oxide with a unique insulator to metal transition (IMT) characteristic, which can be realized by the external excitation of light, heat, or stress^29^. The conductivity of VO_2_ film can be changed by 4–5 orders of magnitude and the phase transition can be completed in the order of sub picosecond^30^. Recently, the active tunable MMA based on IMT properties of VO_2_ has been implemented in other EM frequencies. For example, in the microwave band, Wen *et al*. realized an active tunable MMA based on thermally control by combining VO_2_ with conventional MM resonance and the achieved modulation depth was 63.3%^31^. In the near infrared band, Zhu *et al*. experimentally realized an effective MMA capable of spectral control by minimizing the thermal mass of VO_2_ materials. The device had a tuning range of 360 nm and a modulation depth of 33% at the resonant wavelength^32^. In the infrared band, Kocer *et al*. proposed a thermally tunable MMA with mixed gold-VO_2_ nanostructure arrays^33^. In the optical frequency band, Huang *et al*. designed the Au/VO_2_/Au MM structures and the superior performance of dynamic temperature-controlled optical switch was theoretically and experimentally demonstrated^34^. However, in THz band, the tunable MMA based on IMT characteristics of VO_2_ is still undeveloped.

In this paper, we propose a thermally tunable broadband THz MMA with mixed VO_2_. The designed metadevice is a simple sandwich structure, including symmetrical L-shaped VO_2_ metasurface layer and VO_2_ film ground plane, and these two layers are separated by a thin polyimide (PI) dielectric spacer. The simulation results show that the metadevice is almost transparent to THz wave at low temperature (50 °C) because of the insulation phase of VO_2_ and the maximum absorption is about 11%. With the increasing temperature, VO_2_ gradually transforms from insulation to metal phase, the whole structure is equivalent to a metallic MM structure. When the temperature is over 70 °C, the frequency bandwidth corresponding to absorption above 80% is as wide as 2.0 THz, and the absorption is even close to the perfect absorption of 100% at a specific frequency (1.96 THz). The maximum tunable range of the metadevice can be realized from 5% to 100% by an external thermal excitation. The broadband strong absorption behavior of the proposed metadevice is mainly due to the resonant response of the L-shaped structure and this physical mechanism is analyzed in detail by changing the geometry size of the metasurface structure, intermediate dielectrics, and the VO_2_ ground plane. By simulating the surface electric field distribution of metasurface structure, we prove that the excellent tunability of the absorber is attributed to the IMT characteristics of VO_2_. Meanwhile, we find that the cooling process is less sensitive to the decreasing temperature due to the different conductivity characteristics of VO_2_ materials and the absorber is insensitive to the incident angle variations up to 50°, which is beneficial to practical application over a wide range of incident angle.

## Results

### Hybrid VO_2_ metamaterial absorber

Generally, the typical MMA is a three-layer coupling structure consisting of the metal film mirror, dielectric, and metasurface resonance layers. By designing and optimizing the size, structure, and arrangement of the artificial elements of the MMA, the effective permeability (*μ*_*eff*_) is selected to be equal to the effective dielectric constant (*ε*_*eff*_), and the surface impedance *z*(*w*) of the absorber in a specific frequency band can be adjusted to match the free space, which guarantees that the incident EM waves are hardly reflected. The continuous metal film ground plane is always used to prevent EM waves from penetrating the absorber, so that the transmission is almost zero. Thus, the EM waves can be basically confined to the interior of the MMA until they are completely depleted.

**Figure 1:**
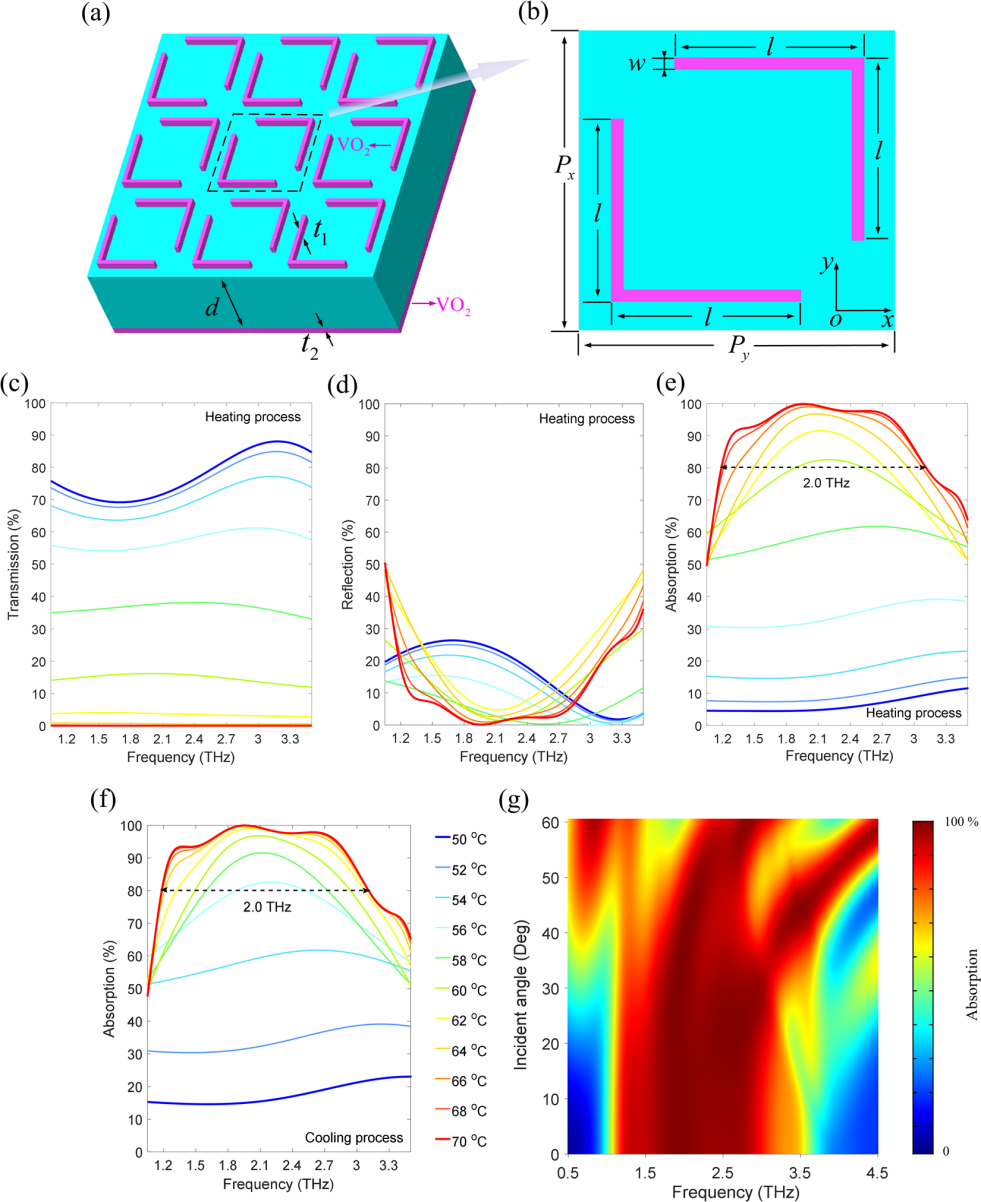
Hybrid VO_2_ metamaterial and broadband tunable THz absorber. (**a**) Schematic diagram of the proposed hybrid VO_2_ MMA, including a middle layer of the PI dielectric spacer (the cyan region) with the thickness *d* = 20 μm, a top layer of the symmetrical L-shaped VO_2_ metasurface structure with the thickness *t*_1_ = 2 μm, and a bottom layer of the VO_2_ ground plate (the purple region) with the thickness *t*_2_ = 4 μm. (**b**) Front view of a unit cell, the geometric parameters are *l* = 30 μm, *w* = 2 μm, *P*_*x*_ = *P*_*y*_ = 50 μm. (**c**–**f**) Simulated THz spectrum of the proposed hybrid VO_2_ MMA at different temperatures for TE mode. (**c**) Transmission. (**d**) Reflection. (**e**,**f**) Absorption for the heating and cooling processes. (**g**) Absorption spectrum of the proposed broadband absorber at the high temperature (70 °C) for different incident angles of THz waves.

Reference to the physical mechanism of the absorber, the THz MMA based on hybrid VO_2_ sandwich structure is proposed and the structural schematic diagram is shown in Fig. 1. The resonant layer in Fig. 1a is composed of a symmetrical L-shaped VO_2_ array with a thickness of *t*_1_ = 2 μm. The lower surface of the MM structure is a layer of VO_2_ thin film with a thickness of *t*_2_ = 4 μm, which is much thicker than the rough 1 μm skin depth of VO_2_ in metal phase around 1.0 THz, which effectively suppresses the transmitted energy of THz waves. The resonant and reflective layers are separated by PI polymer materials with a thickness of *d* = 20 μm. The front view of a unit cell is shown in Fig. 1b and the L-shaped VO_2_ films are symmetrical with respect to the diagonal. Both the lengths of the horizontal and vertical arms of the L-shaped metasurface structure are *l* = 30 μm, the widths are *w* = 2 μm, and the period is *p*_*x*_ = *p*_*y*_ = 50 μm. Because of the unique IMT characteristic of VO_2_, we predict that the designed structure is characterized as a fully permeable THz film at low temperatures due to the insulation phase of VO_2_. While VO_2_ changes from insulation to metal phase at high temperatures, the resonant and reflective layers composed of VO_2_ materials start to work and the whole structure is characterized as a THz absorber. Therefore, based on thermal excitation, we can effectively control the THz absorption characteristics in real time with the assistance of VO_2_ MMs.

With the gradual maturity of material preparation processes and micromachining technology, it is possible to manufacture MM structures in the THz band. So far, based on micro-nano manufacturing technology, THz MM structures with structural unit sizes smaller than tens of nanometers can be successfully manufactured. Therefore, we predict that the designed micron-scale THz MM structure can be easily realized. The continuous VO_2_ film in the structure can be prepared by the sol-gel method, which requires less cost and can be molded at one time. In addition, other preparation methods such as vacuum evaporation, sputtering, and pulse laser deposition (PLD) are sometimes employed. The quality and uniformity of the prepared VO_2_ film can be accurately characterized by using high-resolution X-ray diffraction (XRD), Raman spectroscopy, and Fourier-transform infrared spectroscopy (FTIR). And then by utilizing photolithography and ion beam etching methods, the double L-shaped metasurface resonance structure composed of VO_2_ material can be easily obtained. Here, it is worth noting that compared to most active THz MM devices mixed with multiple materials, the proposed MM sandwich structure can be easily processed, mainly because the structure contains only VO_2_ phase change material and no other functional materials, which effectively simplifies the complexity of the MM structure.

The simulated THz transmission and reflection of the proposed hybrid VO_2_ MMA at different temperatures for heating process are shown in Fig. 1c,d, respectively, and the absorption is shown in Fig. 1e. All the blue and red solid lines represent THz spectra at low (50 °C) and high (70 °C) temperatures, respectively. It can be clearly seen that the THz transmission is above 69% in the whole frequency range at 50 °C, the corresponding reflection is less than 26%, and the absorption is less than 11%. Interestingly, as the temperature increases, the transmission and reflection decrease gradually, which leads to an increase in the absorption. When the temperature increases to 70 °C, the transmission is almost attenuated to zero and the overall reflection is also greatly suppressed, thus an extremely high absorption (greater than 80%) in a continuous range of frequencies with a bandwidth of about 2.0 THz can be obtained, especially at 1.96 THz, a nearly perfect absorption of 100% is achieved. The maximum tunable range of the absorption can be modulated from 5% to 100%. Consequently, we have successfully demonstrated a broadband tunable THz absorber based on thermally control near the room temperature, and the modulation depth is up to 95%, which is difficult to be achieved in the other THz absorbers.

In addition, the THz absorption spectra of the cooling process are also simulated and shown in Fig. 1f. As the temperature decreases gradually, we find that the THz absorption is not attenuated significantly, that is to say, the cooling process is less sensitive to the decreasing temperature. We preliminary predict that the main reason for this fascinating phenomenon is the hysteresis effects of VO_2_ IMT characteristics^29^. When the temperature is 50 °C, VO_2_ is in the insulation phase and fully transparent to the incident THz waves, which results in the high transmission, low reflection and absorption. However, the increasing temperature makes the VO_2_ materials gradually change from the insulation to the metal phase, so the VO_2_ film ground plane gradually suppresses the transmission energy of THz waves, the symmetrical L-shaped metasurface resonant structures attached to the upper surface of the metadevice work on and the incident THz electric fields are gradually coupled into the metal phase VO_2_, which efficiently reduces the reflection. When the temperature increases to 70 °C, VO_2_ is completely transformed into its metal phase and the conductivity is just one order of magnitude lower than that of gold^35^, the incident THz waves can hardly penetrate the metadevice and the transmission is zero. At this time, the resonant response of the L-shaped metasurface structures reaches its maximum, so that the reflection is also significantly weakened, which leads to the strong absorption of the proposed MMA. On the other hand, the conductivity of VO_2_ film is not completely reversible and the similar hysteresis loops during the heating and cooling processes^29^ will lead to the different resonant responses, which is responsible for the device sensitivity to the temperature.

A two-dimensional plot of the absorption spectrum of the oblique THz incident angle on the proposed absorber is depicted in Fig. 1g. The calculated steps of the incident angle and frequency are set to be 1° and 0.001 THz, respectively. It is notable that with the increasing incident angle, the absorption can be maintained at a high level and the absorption bandwidth is also stable for most angles (the red region). The proposed THz absorber is extremely insensitive to the incident angle variations up to 50°, which would be beneficial to practical applications. When the incident angle is beyond 50°, the absorption is obviously attenuated and the absorption bandwidth is narrowed. This is because the strong resonant response of the L-shaped metasurface structure cannot be excited by large oblique incident angle.

### Broadband and perfect absorption

**Figure 2:**
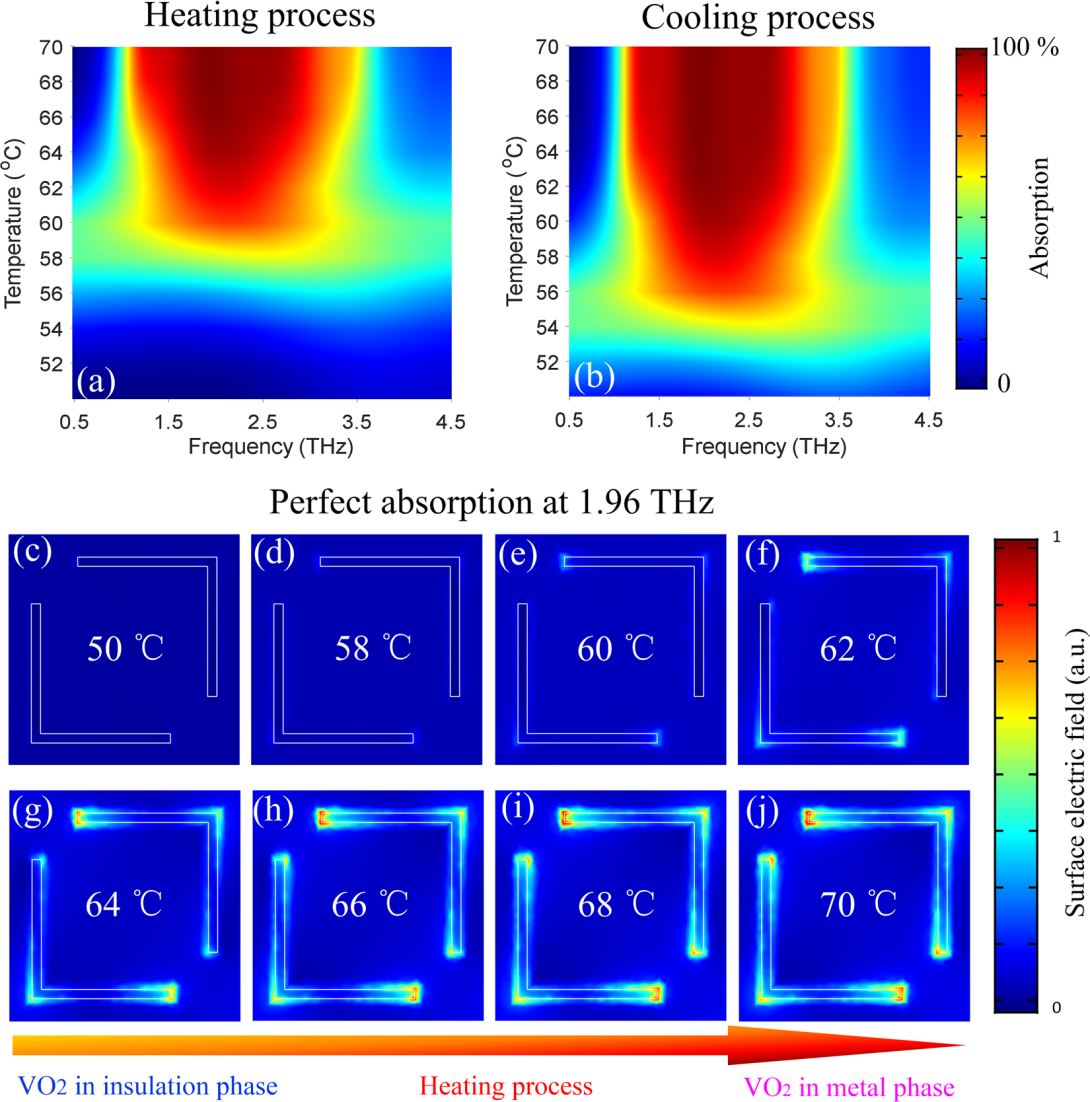
Continuous tuning performance and corresponding physical mechanism. The temperature-dependent THz absorption spectra of the proposed hybrid VO_2_ MMA are depicted in the heating process (**a**) and the cooling process (**b**). (**c**–**j**) Surface electric field distribution corresponding to the perfect absorption at 1.69 THz of the proposed L-shaped metasurface structure for the heating process at different temperatures (50–70 °C).

Although we have just simulated THz absorption spectra at several temperatures in Fig. 1, we can still roughly deduce the tunable behavior of the proposed metadevice by observing the overall trend of the curve variations. To better demonstrate the superior and continuous tuning characteristics of the thermal controlled absorber, a two-dimensional plot of the temperature-dependent THz absorption spectrum in the frequency range of interest is shown in Fig. 2a,b. The calculated steps of the frequency and temperature are 0.001 THz and 1 °C, respectively. Figure 2a illustrates the THz absorption spectra for the heating process from 50 °C to 70 °C. With the gradual increase of temperature, we can clearly see that the absorption does not change significantly (the blue region) until 57 °C, but there is still no obvious resonant absorption in the whole spectrum (the green region) until 60 °C, the strong THz absorption band is excited and the bandwidth is gradually broadened with the increasing temperature (the red region). When the temperature gets 68 °C, the absorption bandwidth is increased to about 2.0 THz and reaches saturation.

Meanwhile, to clearly describe the tunable characteristic of the absorber during cooling process, a temperature-dependent two-dimensional graph is also depicted in Fig. 2b, which is quite different from that of the heating process. We find that with the decrease of temperature, the absorption does not change obviously (the red region) until 64 °C. With the further decreasing temperature, the absorption and its bandwidth gradually become small and narrow, respectively. When the temperature drops to 56 °C, the resonant absorption disappears as shown by the green region. The simulated results more clearly demonstrate that the modulations of THz absorption are different in the heating and cooling processes and the sensitive temperature in the cooling process is lower. The temperature-dependent two-dimensional plots unambiguously indicate a drastic but continuous spectrum tuning behavior before saturation for the heating and cooling processes. Based on the detailed analysis above, with the assistance of the IMT characteristic of VO_2_ materials, the superior performance of the proposed broadband tunable THz absorber has been well demonstrated and the achieved condition of thermal excitation is close to room temperature, which greatly promotes the practical application of the designed metadevice.

It is well demonstrated that the proposed broadband absorber can achieve active tunability of wide range with the assistance of the VO_2_ phase change material. To further study the physical mechanism of the achieved tunable behavior based on temperature control, the surface electric field distribution for heating process at different temperatures is simulated and the results are shown in Fig. 2c–j. It can be found that there is almost no electric field distribution on the surface of L-shaped structure at 50 °C, indicating that the incident THz waves almost completely penetrate the MM structure. This is because VO_2_ exhibits insulation characteristics and is transparent to THz waves at low temperature. The ground plane and L-shaped layer composed of VO_2_ have no effect on THz waves and result in the low absorption. With the increase of temperature from 58 °C to 68 °C, the electric field is coupled to the two ends of the VO_2_ arms and gradually diffused to the surface of the whole arm and the cross area because VO_2_ is gradually transformed from the insulation to metal phase. The L-shaped resonant response and the reflection of VO_2_ ground plane are enhanced simultaneously, which results in a sharp increase in the absorption. In addition, we find that the surface electric field distributions at 68 °C and 70 °C are almost the same. Because VO_2_ is completely transformed into its metal phase at 68 °C, the resonant response induced by the VO_2_ metasurface structure will not be further enhanced with the increasing temperature. Therefore, based on external heating excitation, the proposed hybrid VO_2_ MM structure can realize the alternation between the fully transparent thin film and the broadband absorber in THz band.

Here, besides the dramatic manipulation of the response, it is important to emphasize that since the IMT phase transition time of the VO_2_ material can be completed in sub-picoseconds, our proposed broadband tunable THz absorber based on hybrid VO_2_ MMs can achieve ultra-fast dynamic response under external thermal excitation. The capable of offering ultrafast modulation of THz radiation is difficult to achieve in other THz modulators. The proposed hybrid metamaterial concepts may open up new avenues for highly tunable ultrafast THz devices.

### Absorption mechanism and performance

**Figure 3:**
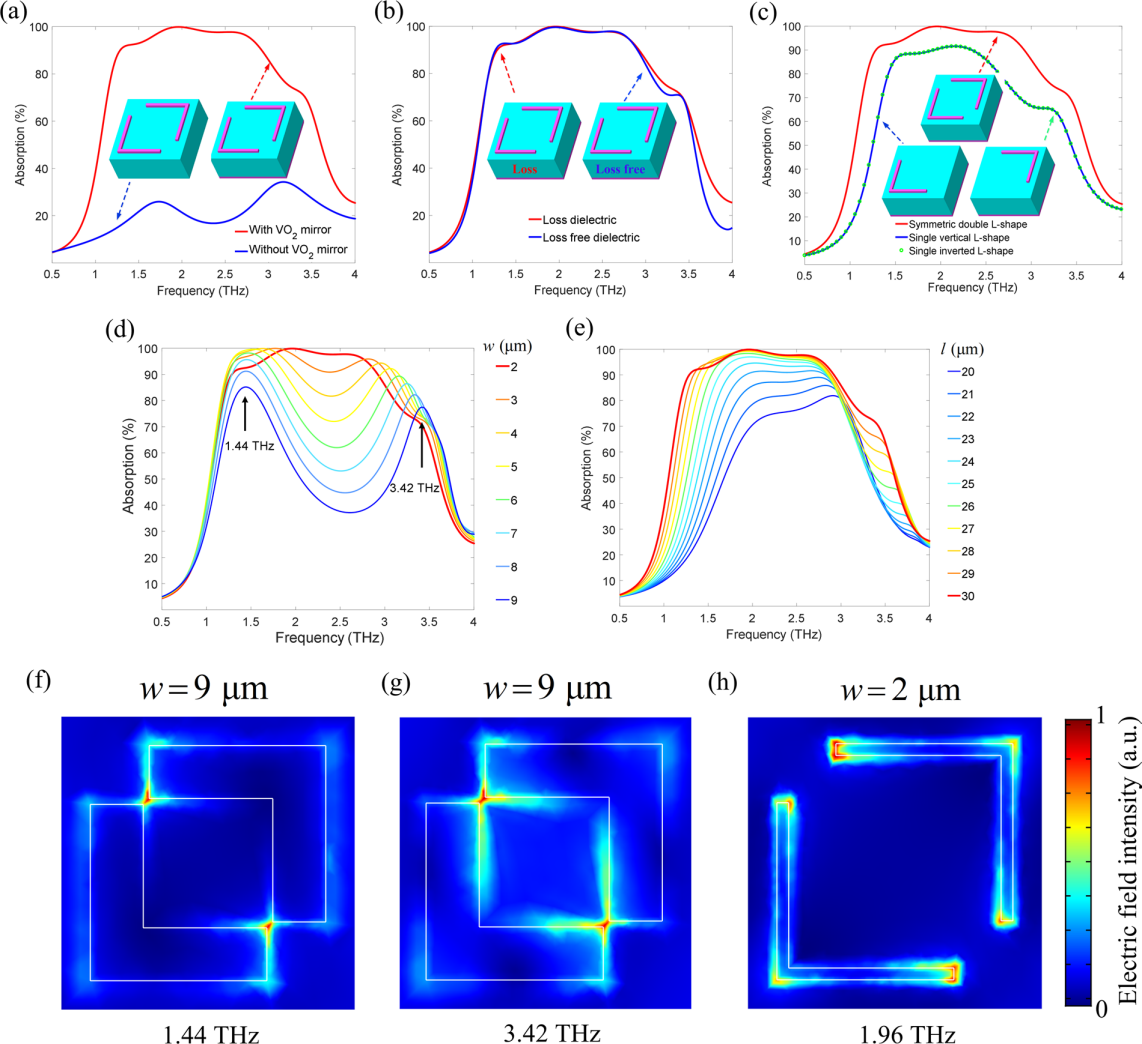
Effects of MM structure on broadband absorption performance. (**a**–**e**) Absorption spectra of the proposed hybrid VO_2_ MMA at high temperature (70 °C) with and without VO_2_ gound plane (**a**), with different dielectric layers (loss and loss free) (**b**), with different L-shaped metasurface structures (**c**), with different arm lengths from 20 μm to 30 μm (**d**), and with different arm widths from 2 μm to 9 μm (**e**). (**f**–**h**) Surface electric field distribution corresponding to the resonance absorption frequencies of 1.44 THz and 3.42 THz with *w* = 9 μm, and of 1.96 THz with *w* = 2 μm, respectively.

To investigate the physical mechanism of the broadband absorption characteristic of the proposed absorber at high temperature, it is necessary to clarify the contribution to THz absorption of each layer in the designed sandwich structure. Here, the VO_2_ ground plane in metal phase is used as a mirror to enhance absorption. As shown in Fig. 3a, it can be clearly seen that the achieved maximum absorption without VO_2_ film substrate in the whole spectrum is only 33%, much smaller than that with VO_2_ plane, which fully confirms the role of VO_2_ mirror. In addition, the effect of dielectric loss (PI) in the middle layer on THz absorption is also studied and the simulation results of THz absorption spectra for different dielectrics (loss and loss free) are depicted in Fig. 3b. We find that there is no obvious change in absorption and bandwidth. So, the dielectrics have the limited effect on the absorption. Unlike the other absorbers^14,36^, the insensitivity of dielectric layers in the proposed one might be significantly applicable in many fields.

The single vertical and inverted L-shaped resonance structures are also designed to study the physical mechanism of the broadband absorption. The simulated results are shown in Fig. 3c. It can be seen that the two absorption spectra almost coincide due to the symmetry of the structure. However, the spectrum bandwidth with the absorption above 80% is only about 1.0 THz, which is much narrower than that of double L-shaped (2.0 THz). And the strongest absorption is still less than 90%. So, the double L-shaped structures can enhance the resonant response and lead to the strong enough absorption.

Through the detailed analysis above, it can be firmly believed that the energy of incident THz waves is mainly dissipated by the symmetrical L-shaped VO_2_ metasurface resonance layer. To further demonstrate the strong absorption induced by the L-shaped structure, we simulated the THz absorption spectra with different geometric parameters. Firstly, the dependence of the absorption spectra on the width *w* is simulated and shown in Fig. 3d. It is surprising to find that with the increasing width from 2 μm to 9 μm, the broadband absorption spectrum is destroyed and gradually splits into two strong resonant peaks near 1.44 THz and 3.42 THz, illustrating that width has a great impact on the resonances between the L-shaped structure and the THz radiation. However, from other points of view, the THz absorber with dual resonance absorption channel also has important development prospects in practical applications.

As another important geometry parameter of the L-shaped structure, the influence of arm’s length on absorption is also studied. The absorption spectra of the arm length changing from 20 μm to 30 μm are shown in Fig. 3e. With the increasing arm length, the absorption spectrum is broadened gradually and the absorption is also enhanced. This is because the L-shaped resonant characteristic is mainly due to the electric field coupled to the surface of the arms. In addition, we find that the increased arm length leads to a red shift in the absorption spectra. It can be explained that the different resonant modes have been produced by changing the size of the structure. The arm length is inversely proportional to the resonant frequency^37^. With the increasing arm length, the resonance absorption band will accordingly shift to the low frequency range. Consequently, it can be concluded that the geometrical size and the numbers of L-shaped structures will have a great impact on the THz absorption. The optimal size of the double L-shaped structure can provide the high-performance THz absorber.

To better understand the physical mechanism of the absorption spectrum varying with the width of L-shape, the surface electric field distribution is simulated and shown in Fig. 3f–h. The electric field distributions corresponding to the resonance frequencies of 1.44 THz and 3.42 THz are illustrated in Fig. 3f,g, respectively, when the width is *w* = 9 μm. It can be clearly observed that the two arms of the L-shaped structure are very close to each other, resulting in the strong dipole resonance (1.44 THz) at both ends of the arm. The obvious difference can be found that the electric field corresponding to the resonant mode at high frequency (3.42 THz) is not only distributed at both ends of the arms, but also at the edges of the arms. As the width decreases, the two ends of the arms are gradually separated, which weakens the dipole resonance, so the dual resonance absorption mode is suppressed gradually. When the width is reduced to 2 μm, the two L-shaped structures are completely separated. By observing the electric field distribution at the perfect absorption frequency of 1.96 THz shown in Fig. 3h, we find that the electric field is fully coupled to the whole surface of the two L-shape structures, which leads to the broadband and high THz absorption spectrum.

**Figure 4:**
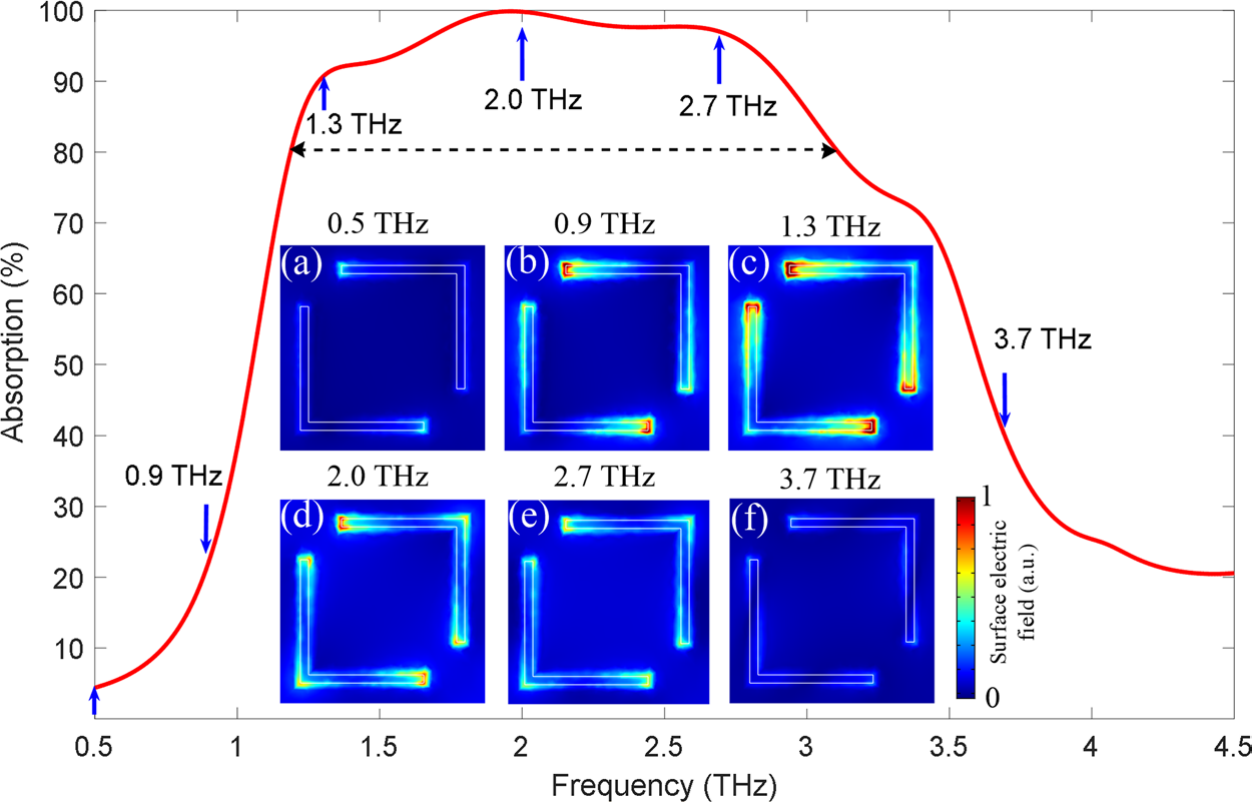
Physical mechanism of broadband absorption. (**a**–**f**) Surface electric field distributions of the L-shaped VO_2_ metasurface structure in the proposed broadband absorber at different frequencies of 0.5 THz, 0.9 THz, 1.3 THz, 2.0 THz, 2.7 THz, and 3.7 THz. The dashed line denotes the absorption bandwidth of about 2 THz.

To clearly show the different resonance characteristics of the L-shaped metasurface structures in the whole absorption spectrum, we simulated the surface electric field distribution corresponding to different absorption frequencies and the results are presented in Fig. 4. We find that the absorption at 0.5 THz is only 5%, as shown in Fig. 4a. There is almost no electric field distribution on the surface of the structure, indicating that the resonant response does not occur. The MMA does not interact with the incident THz waves, which leads to the extremely low absorption. When the frequency gradually shifts to 0.9 THz, the absorption is increased to 22%. This is because a weak resonant response is generated in the L-shaped structure and the electric field is coupled at both ends of the arms as shown in Fig. 4b. And the absorption is also increased. We have simulated the surface electric field distribution corresponding to the strong resonance absorption frequencies of 1.3 THz, 2.0 THz, and 2.7 THz with the absorption above 80%, and the results are shown in Fig. 4c–e, respectively. Obviously, the electric field is strongly coupled to the whole surface of the L-shaped structure, including two ends of the arms, arm edges, and the cross areas. In these cases, the resonant responses are very strong and the absorptions are also intense. Notably, when the frequency shifts to 3.7 THz, away from the frequency band with high absorptions, the coupled electric field intensity of the L-shaped surface is greatly reduced in Fig. 4f, indicating that the resonant response disappears and the absorption is also decreased. Therefore, by simulating the surface electric field distributions at different frequencies, we have clearly demonstrated that the L-shaped resonant structure is entirely responsible for the broadband and strong absorption behavior of the proposed MMA.

So far, researchers have proposed various approaches to modulate THz waves in broadband. For example, hybrid two-dimensional materials such as graphene THz broadband absorbers have been widely proposed^38–42^. However, due to the limited tunability of the conductivity of graphene itself, which greatly limits the modulation depth and tunable range of the device. Moreover, the conductivity of graphene can be effectively modulated by the applied bias voltage, so most of the reported THz devices contain electrode structures, which greatly increases the difficulty of processing. At the same time, the electronically controlled modulation method cannot achieve non-contact tunability, which limits the practical application of the device to some extent. The proposal of photo-excited broadband tunable THz absorber effectively solves this problem^43–45^, but because of the limited spot size of the external pump light and the uncontrollable transmit power, this device cannot effectively regulate large area THz radiation. Recently, in the THz band, a novel tunable broadband device based on coded metasurface has been reported^46–49^. Through the real-time programmable design of the metasurface structure, the amplitude, phase and polarization of THz wave can be effectively regulated, but the complex structure increases the processing difficulty of the device. In addition, it is well known that, compared with other types of modulators, the mechanically tunable THz devices were first proposed^50,51^, its advantages lie in enabling strong tunable capacity, avoiding dynamic decay in resonance strength, and maintaining Q factors during tuning. However, the THz devices that utilize mechanical stimulation for dynamic tuning, have received much less attention, mainly because of the more complicated fabrication process and the relatively slow response time of mechanical systems.

Different from all methods in the past, our proposed broadband tunable THz absorber based on hybrid VO_2_ MMs can achieve a higher modulation depth at room temperature. Considering the hysteresis behavior of VO_2_ materials and the IMT phase transition time on the order of picoseconds, the proposed THz device exhibits a strong memory effect and has an ultra-fast response time. At the same time, unlike most active THz MMs devices, the structure we designed contains only one VO_2_ material in addition to the dielectric, so it is easy to process.

## Discussion

Based on the IMT characteristic of VO_2_, we have proposed a broadband tunable THz MMA. At low temperature (50 °C), the designed MM structure has lower absorption of incident THz waves due to the insulation phase VO_2_. However, with the increasing temperature from 50 °C to 70 °C, the VO_2_ film gradually transforms from the insulation to metal phase, at this time, the proposed structure gradually transforms into a broadband THz absorber. An extremely high absorption (greater than 80%) in a continuous range of frequencies with a bandwidth of about 2.0 THz has been obtained. Especially at 1.96 THz, a nearly perfect absorption has been achieved. The maximum tunable range of the absorption has been realized from 5% to 100% by external thermal excitation. Benefiting from the phase-change VO_2_ material, the applied external thermal excitation is close to room temperature, which plays an important role in promoting the practical application of the metadevice. The large dynamic range and convenient thermal control are impossible to be achieved in other absorbers. Meanwhile, we have also demonstrated that the proposed THz absorber is extremely insensitive to the incident angle variations up to 50°. We believe that the concept of the proposed THz device could be readily extended to other frequency regimes. Such a thermally tunable broadband THz absorber may be applied in various fields, such as imaging, cloaking, modulating, and filtering.

## Methods

The full-wave EM simulations are implemented with the finite element solution by using the commercial software package of COMSOL Multiphysics. The open boundary is applied along the *z*-direction, which is upward and normal to the metasurface. To simulate the infinite periodic array structure, the periodic boundary conditions along the *x*-*y* plane (*x* and *y* directions) are performed. The incident THz wave is selected as a TE mode and perpendicular to *x*-*y* plane. To ensure the accuracy of the calculation results, the ultra-fine grids are adopted and the frequency step is set to be 0.001 THz. The dielectric constant of the PI is 2.88–0.09i. The IMT characteristic of VO_2_ in THz band needs to be described by the Bruggeman effective medium theory (EMT). The dielectric function *ε*_*C*_ can be expressed as^52^

1 $${\varepsilon }_{C}=\frac{1}{4}\{{\varepsilon }_{D}(2-3{f}_{v})+{\varepsilon }_{M}(3{f}_{v}-1)+\sqrt{{[{\varepsilon }_{D}(2-3{f}_{v})+{\varepsilon }_{M}(3{f}_{v}-1)]}^{2}+8{\varepsilon }_{D}{\varepsilon }_{M}}\},$$

where *f*_*v*_ is the volume fraction of the metal component, *ε*_*D*_ and *ε*_*M*_ are dielectric functions of VO_2_ thin films in insulation and metal phase, respectively^29^. In addition, the functional relationship between the fraction *f*_*v*_ and the temperature *T* can be described by the Boltzmann function,

2 $${f}_{v}={f}_{{\rm{\max }}}(1-\frac{1}{1+\exp [(T-{T}_{0})/{\rm{\Delta }}T]}),$$

where *T*_0_ is the phase transition temperature, Δ*T* is the transition width, and *f*_max_ is the maximum volume fraction. By combining Eqs (1) with (2), the conductivity of VO_2_ thin films corresponding to different temperatures in the phase transition process is expressed as^35^

3 $$\sigma =-\,i{\varepsilon }_{0}\omega ({\varepsilon }_{C}-1).$$

The reflection and transmission of the proposed MMA can be acquired by simulating the complex frequency-dependent *S* parameters (*S*_11_ and *S*_21_), so the absorption coefficient *A* can be calculated by^53^

4 $$A=1-{R}_{s}-{T}_{s}=1-{|{S}_{11}|}^{2}-{|{S}_{21}|}^{2},$$

where *R*_*s*_ = |*S*_11_|^2^ and *T*_*s*_ = |*S*_21_|^2^ are the reflection and transmission coefficients, respectively.
